# Investigation of the leaching behavior of Na and Si in simulated HLW borosilicate glass obtained from the waste of a 1000 MWe class PWR reactor: using the response surface method

**DOI:** 10.3389/fchem.2024.1349531

**Published:** 2024-03-25

**Authors:** Mohammad Hosseinpour Khanmiri, Ali Yadollahi, Mohammad Samadfam, Hamid Sepehrian, Mohammad Outokesh

**Affiliations:** ^1^ Department of Energy Engineering, Sharif University of Technology, Tehran, Iran; ^2^ Nuclear Fuel Cycle Research School, Nuclear Science and Technology Research Institute, AEOI, Tehran, Iran

**Keywords:** borosilicate glass, nuclear waste glass, PCT test, leaching rate, HLW immobilization, response surface methodology, modeling

## Abstract

The immobilization of high-level nuclear waste (HLW) in glass waste matrices provides the key safety function of slowing down radionuclide emissions from an underground disposal site. This study examines the leaching behavior of two major elements, Na and Si, in HLW borosilicate glass simulated from waste of a 1000 MWe class pressurized water reactor (PWR) using response surface methodology and Box-Behnken Design. The design of the experiment was carried out considering three independent variables: the pH of the solution, the contact time, and the leaching temperature, leading to 17 leaching runs performed using the static product consistency test (PCT). The results of statistical analysis (ANOVA: analysis of variance) indicated that the effects of the individual variables and the interactions between them were statistically significant, and the relative consistency of the data further confirmed the model’s applicability. Data obtained from the PCT experiments revealed that the leaching behavior of Na and Si in the evaluated waste glass exhibited similar behavior to previously researched glasses for each condition tested.

## Highlight

• The leaching behavior of Na and Si as a function of time, pH, and temperature in HLW borosilicate glass using the RSM approach was examined.
• The 3D surface plots showed that with the simultaneous increase in pH and temperature over 14 days, the leaching rates of Na and Si increased.
• At 70 ℃ with an increasing pH, the leaching rates of Na and Si increased, but the leaching time had a little negative effect on the rates.
• The pattern of changes in the leaching rates of the elements studied in this work and in other different waste glasses is very similar.
• The experimental validation of the BBD model showed that the actual leaching rates are reasonably close to the predicted values and are located within 95% PI low and 95% PI high intervals.

## 1 Introduction

The immobilization of high-level radioactive waste (HLW) materials is thought to be the most important step in the final phase of radioactive waste management technology (Ewing et al., 2004; Weber et al., 2009; Ojovan and Lee, 2011; McCloy and Goel, 2017; Hosseinpour Khanmiri and Bogdanov, 2018; Hosseinpour Khanmiri et al., 2018; Rahman and Saleh, 2018; Hyatt and Ojovan, 2019; Ojovan and Yudintsev, 2023; Hosseinpour Khanmiri et al., 2024). Most of the available data is related to the development of materials for the long-term storage or disposal of high-level nuclear waste materials, either from the reprocessing of spent commercial reactor fuels or from a number of defense reprocessing operations. These analyses use a modular strategy to take into account the time-dependent development of technical barriers as well as the dynamic character of biological and hydrological processes in the host environment (Rahman et al., 2014). In order to reduce the likelihood of radioactive transportation or dispersion during the operation and disposal stages of the waste lifecycle, radioactive waste must be immobilized by embedding, solidification, or encapsulation. Waste is immobilized by chemically incorporating it into the framework of a compatible matrix. Its primary safety features are ensuring structural integrity, resisting deterioration, and limiting water infiltration and radioactive leakage. Stabilizing radioactive waste involves using methods like cement, ceramic, polymer, and glass solidification (Yim and Linga Murty, 2000; Lee et al., 2013; Rahman et al., 2013; Ojovan et al., 2019). Out of all of them, vitrified forms are widely accepted as the most feasible and stable form for disposing HLW. According to the obtained laboratory data and due to the special structure and chemical composition, the glass waste matrices have shown acceptable resistance to maintaining their elements in simulated water environments, so these materials provide the key safety function of slowing down radionuclide emissions from an underground disposal site (Ebert and Jerden, 2019).

Despite the fact that a wide variety of ceramic materials and glass have been considered potential candidates for the immobilization of HLW, borosilicate glass is currently the most widely used wasteform. Due to this choice, borosilicate glass is currently being used as the host for the immobilization of HLW in a number of industrial vitrification facilities across the globe (Kaushik et al., 2006; Ojovan and Lee, 2011; Stefanovsky et al., 2017). The borosilicate glass’s flexibility in terms of waste loading and capacity to incorporate a variety of waste elements, in addition to its strong glass-forming capabilities, mechanical integrity, chemical resistance, and superior thermal and radiation stability, are the reasons for this decision (Manaktala, 1992; Ojovan et al., 2019). Since water is the most prevalent substance in many environments, borosilicate glass researchers from several fields are very interested in how water interacts with glass. The three phases that make up the interaction between glass and water are the diffusion of water molecules into the glass structure, ion exchange with protons, and hydrolysis of network-modifying species in the glass structure (Wu et al., 2011; Gin et al., 2015a). Whenever waste borosilicate glass is exposed to a natural environment, such as groundwater that is either flowing or still, chemical reactions start at the surface of a waste glass, and then the entire waste glass is affected by these interactions, based on its composition, the pH of the solution, the contact time, and the ambient temperature. As a result, by studying the leaching behavior of the major elements of the numerous borosilicate glasses depending on various factors (temperature, pH, time, etc.) via kinetic models, it is feasible to forecast glass durability and build glasses that adhere to certain leaching requirements based on short-term tests, as well as forecast the long-term dissolution behavior of glasses. In previous work (Kim et al., 2011), the temperature and pH dependences of four glasses created by the KHNP (the Korea Hydro & Nuclear Power Co., Ltd.) on the leaching behavior were discovered by utilizing a collection of pH buffer solutions with a pH range of 4–11 at temperatures of 40, 70, and 90°C over 3 weeks via the MCC-1 leaching standard test (ASTM C1220). When the temperature ranged from 40°C to 90°C and the leachant condition ranged from pH 4 to pH 11, all of the test glasses had a forward dissolve rate of ≤10 [g/(m^2^. d)]. In a later paper (Gin et al., 2015b), at pHs of 9.0 and 11.5 thorough static leaching tests on international simple glass (ISG) were conducted and verified the growth of alteration layers on the glass surface as a result of the release of weakly bonded cations like Ca^2+^, Na^+^, and boron species. Ebert and Jerden (Ebert and Jerden, 2019) reported results of modified ASTM C1285 tests performed at 90°C using AFCI (Advanced Fuel Cycle Initiative) and LRM (low-activity reference material) glasses to ascertain if dissolving rate dependencies on pH, Al, and Si contents must be taken into account. They came to the conclusion that the pH, Si, and Al concentrations, as well as maybe other facets of the glass composition, are probably what ultimately cause the resumption rate to occur. Here, it is better to briefly mention that the alteration of radioactive waste glass in contact with water can include the following stages: I. Initial diffusion or interdiffusion (exchange between glass network-modifying cations and protons in solution). II. The initial or forward rate (hydrolysis of the glass network). III. The rate drop (saturation of the solution with silicon concentration and formation of gel on the surface of the glass). IV. The residual rate (in a closed system with secondary phase precipitation, the leaching rate remains at a relatively low but approximately constant rate). V. A possible resumption of alteration in particular conditions (precipitation exceeding the pH threshold, self-sustaining precipitation, destabilizing the gel, and the resumption rate of glass alteration) (Frugier et al., 2008). In the study (Neeway et al., 2019), using single-pass flow-through (SPFT) testing at a temperature of 90°C in buffered solutions of pH (RT) 4, 7, 9, and 11 as well as the static product consistency test (PCT), the corrosion behavior of oxyapatite [Ca_2_Nd_8_(SiO_4_)_6_O_2_] and powellite [(Ca,Sr,Ba)MoO_4_] in glass-ceramic nuclear waste materials was examined. The findings showed that the individual phases’ dissolution kinetics varied between pH investigations. According to the material presented above, it can be easily concluded that it is necessary to examine the factors of temperature, pH, and time as leaching criteria to analyze and optimize the resistance of nuclear waste glass. For this purpose, different leaching test methods for waste glasses allow experts in the field of nuclear waste to compare and optimize the resistance of the glass. In the meantime the simultaneous investigation of the effect of several independent variables (temperature, pH, time, and.) on a dependent variable (leaching rate), interesting and promising results will be produced.

In the present study, the dissolving kinetics of non-radioactive high-level waste borosilicate glass simulated from waste of a 1000 MWe class PWR reactor were experimentally investigated. For this purpose, the short-term leaching behavior of Si and Na elements was studied by using a series of pH buffer solutions at different temperatures for various time periods. The leaching process was assessed by conducting a product consistency test method B (PCT-B) (Jantzen et al., 1992; Crawford et al., 2007; Jantzen and Bibler, 2009; Vienna et al., 2013) in accordance with American Society for Testing Materials (ASTM) Standard C1285. To investigate the effect of leaching parameters, including the pH of the solution, the contact time, and the temperature of the environment, Response Surface Methodology (RSM) with a Box-Behnken Design (BBD) implemented in the Design-Expert software was utilized, producing a three-dimensional response surface.

## 2 Experimental

### 2.1 Glass specimen

A non-radioactive high-level waste borosilicate glass simulated from waste of a 1000 MWe class PWR reactor (PWRHLW-BSG-1) was employed as a glass specimen in this study. The maximum non-radioactive waste loading in borosilicate glass is 26.25 percent. The corresponding sample was obtained from the Nuclear Fuel Cycle Research School, Nuclear Science and Technology Research Institute, Atomic Energy Organization of Iran. The chemical composition of PWRHLW-BSG-1 is shown in Table 1. The chemical composition of borosilicate glass is similar to the composition of general borosilicate glass and the chemical composition of nuclear waste was simulated from waste of a 1000 MWe class PWR reactor. PWRHLW-BSG-1 was synthesized in a laboratory electric furnace (F11L-1250, Iran) at a temperature of 1,200°C for 2 h. Clean crushed waste glass of a particle size between 149 μm and 74 μm (−100 to +200 mesh) was utilized for the leaching test. The sample was milled under the following conditions: laboratory milling machine: Sanat Ceram, FMSV, 68 kg, 375 W; the volume of mill/jar: 500 cc; characteristics of balls: 5 alumina balls each with a diameter of 3 cm; rotation speed: 400 rpm; time of milling: 1 h; additives: without any additives; the state of the sample during milling: completely dry; homogeneous sample size in the milling process: 4 pieces of approximately 0.8 cm^3^; laboratory sieves: 100 and 200 mesh. Specific surface areas of the finely powdered sample, determined by the Brunnauer-Emmett-Teller (BET) method (NOVA 2200e, Quantachrome), were 1.97 10^−2^ m^2^/g. To determine the specific surface area in this analysis, 2 g of clean crushed waste glass of a particle size between 149 μm and 74 μm were washed with distilled water to remove any foreign contamination and dried in an oven at 60°C for 24 h. Before determining the specific surface area, the powder sample are degassed at a temperature of about 250 ℃ for 4 h under vacuum. Using the Archimedes technique, the density of the PWRHLW-BSG-1 specimen was measured and found to be 2.737 g cm^−3^.

**TABLE 1:** Chemical composition of PWRHLW-BSG-1 simulated waste glass.

Oxide	Weight %	Oxide	Weight %	Oxide	Weight %
Al_2_O_3_	4.57	Y_2_O_3_	0.26	Nd_2_O_3_	3.15
B_2_O_3_	13.20	ZrO_2_	3.15	La_2_O_3_	1.25
CaO	3.84	MoO_3_	2.23	Fe_2_O_3_	3.68
Li_2_O	1.84	MnO_2_	0.46	NiO	0.52
Na_2_O	5.16	Ag_2_O	0.04	Cr_2_O_3_	0.66
SiO_2_	42.78	CdO	0.05	P_2_O_5_	0.34
ZnO	2.36	SnO_2_	0.05	Na_2_O	5.25
SeO_2_	0.29	BaO	0.79	Total	100
Cs_2_O	1.40	Ce_2_O_3_	1.58		
SrO	0.45	Pr_2_O_3_	0.66		

### 2.2 Experimental design for the leaching of simulated waste glass

The Design-Expert software (version 12) from Stat-Ease, Inc. was employed for the experimental design. For this purpose, the investigated parameters were modeled, employing the Box-Behnken Design (BBD) approach in response surface methodology (RSM). To evaluate the leaching behavior of Si and Na elements in the PWRHLW-BSG-1 simulated waste glass, three independent variables, the pH of the solution, the contact time, and the leaching temperature, leading to 17 leaching runs, were generated, as shown in Table 2. Experiments using the PCT leaching test were conducted at three different temperatures of 50, 70, and 90°C (Factor A), under alkaline conditions at pH (Factor B) 8, 10, and 12, and during periods of 7, 14, and 21 days (Factor C). In order to assess the data’s statistical significance, analysis of variance (ANOVA) was used, taking into account *p* ^1^ < 0.05, Eq. 1 (Rodrigues et al., 2009; Kweinor Tetteh et al., 2021):

$$NE=K^{2}+K+Cp$$ (1)

**TABLE 2:** Experimental design matrix (from BBD) for the leaching of Si and Na in the PWRHLW-BSG-1.

Run	Factor A: Temp ℃	Factor B: pH	Factor C: Time, d
1	70	8	21
2	50	8	14
3	70	10	14
4	50	10	21
5	70	12	7
6	70	12	21
7	50	12	14
8	90	10	21
9	70	10	14
10	90	12	14
11	70	10	14
12	90	10	7
13	70	10	14
14	50	10	7
15	90	8	14
16	70	8	7
17	70	10	14

Here, *NE*, *K*, and *Cp* stand for, respectively, the total number of experiments, the number of factors, and the number of repeated center points. The polynomial quadratic represented in Eq. 2 was used to examine the pivotal relationship between the independent and dependent variables.

$$Y=\beta_{0}+\sum_{i=1}^{k}\beta_{i}x_{i}+\sum_{i=1}^{k}\beta_{ii}\overset{2}{\underset{i}{x}}+\sum_{i=1}^{k-1}\sum_{j=2}^{k}\beta_{ij}x_{i}x_{j}+\varepsilon$$ (2)

Here, *Y* is a response variable, *β*_*0*_ is a constant, *k* is the number of independent variables, *β*_*i*_ is the regression coefficient, *x*_*i*_ and *x*_*j*_ are the independent variables in coded levels and *ε* is the unidentified error constant that has a mean of zero as a random experimental error.

To provide more experimental data for a wider comparison and to check the accuracy of the results related to the leaching behavior of Si and Na, six additional tests described in Table 3 were performed in parallel with the 17 tests provided in Table 2.

**TABLE 3:** Excess experiments other than leaching runs of BBD for confirmation of the leaching of Si and Na in the PWRHLW-BSG-1.

Test	Temp ℃	pH	Time, d
1	70	8	14
2	70	10	7
3	50	10	14
4	90	10	14
5	70	10	21
6	70	12	14

### 2.3 PCT leaching test

**FIGURE 1:**
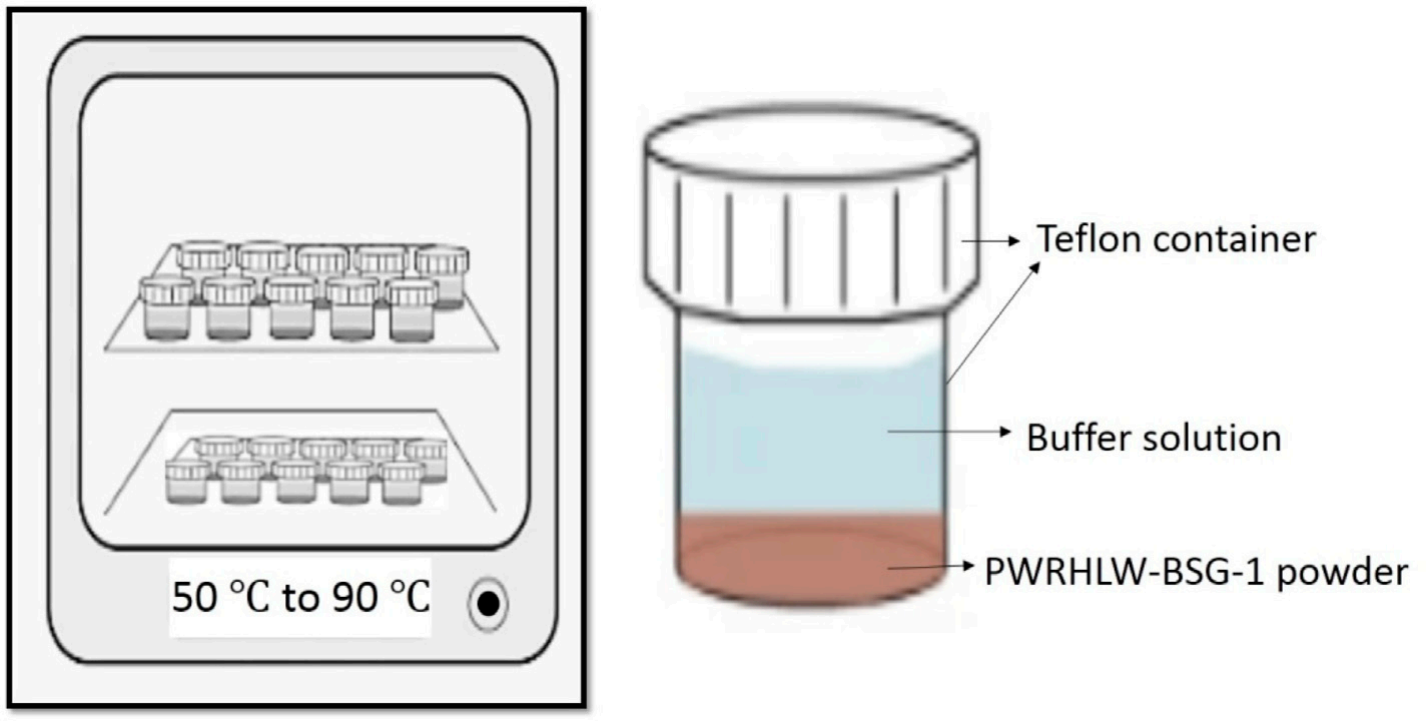
PCT-B test in accordance with ASTM—C1285.

PCT-B on PWRHLW-BSG-1 was carried out using 23 Teflon test containers, each having a capacity of 100 cm^3^. The amount of crushed glass in each vessel was precisely 1.5 g. The alkaline buffer solutions at pH 8, 10, and 12 were utilized to control the pH during PCT tests. In order to create buffer solutions with pH values of 8 and 10, determined quantities of the organic tris hydroxymethyl aminomethane buffer (THAM, CAS-No: 77-86-1, Merck KGaA, 64271 Darmstadt, Germany) were added to distilled water, reaching approximately 0.05 M concentration, and adjusted to the required pH at room temperature with 15 M HNO3. A buffer solution with a pH of 12 was created by preparing 0.01 M LiOH and 0.01 M LiCl and adjusting with 0.01 M LiOH. According to the experimental design, a 15 cm^3^ buffer solution with different pH values was added to each vessel. The ratio of the waste glass sample surface area to leachate volume (S/V) was 1,970 m^−1^. The Teflon test containers were kept in ovens set at 50, 70, and 90°C for durations of 7, 14, and 21 days (Figure 1). The containers were taken out of the oven at the conclusion of each test and left to cool. After that, the leachates were filtered to determine the pH values. Table 4 indicates the pH values of the leachate of every sample before and after the leaching test. Finally, the leachate of each waste glass specimen was analyzed to measure values of Si and Na elements using the inductively coupled plasma atomic emission spectrometry technique (ICP-AES) (PerkinElmer OPTIMA 2000).

**TABLE 4:** The pH values of the leachates before and after the PCT leaching test for BBD and six excess experiments runs.

Run	Initial pH value	Final pH value	Temp ℃	Time, d
1	8	6.29	70	21
2	8	7.79	50	14
3	10	9.68	70	14
4	10	9.44	50	21
5	12	9.80	70	7
6	12	9.66	70	21
7	12	10.90	50	14
8	10	8.45	90	21
9	10	9.71	70	14
10	12	10.30	90	14
11	10	9.66	70	14
12	10	8.51	90	7
13	10	9.65	70	14
14	10	9.56	50	7
15	8	6.07	90	14
16	8	7.74	70	7
17	10	9.69	70	14
1	8	7.75	70	14
2	10	9.46	70	7
3	10	9.68	50	14
4	10	9.67	90	14
5	10	—	70	21
6	12	10.65	70	14

### 2.4 Quantitative description of dissolution rates

The effectiveness of the radioactive immobilization approach is assessed by the rate at which radionuclides can escape from the wasteform during long-term storage. Leach rates are the most important factors in determining how efficiently glass can retain radioactive components since water is the most likely means for radiation to enter the biosphere once more. Typically, the amount of each glass constituent released into the solution when the glass comes into contact with it is measured in order to determine the rate of glass dissolution. The characteristics of the contact solution and the chemical makeup of the glass determine the rate at which the constituents are released. The normalized leaching rate (*NLR*_*i*_) for a specific element (i) from the waste glass has been computed on the basis of the following Eq. 3 (Committee, 2002; Ojovan and Lee, 2011; Inagaki et al., 2012; Thorat et al., 2019):

$${NLR}_{i}=\frac{C_{i}}{\left(SA/V\right)\left(t\right)\left(f_{i}\right)}$$ (3)

Here, *NLR*_*i*_ [g/(m^2^. d)] is the normalized leaching rate of element (*i*) from the waste glass; *C*_*i*_ (g/m^3^) is the concentration of element (*i*) in the solution; *SA* (m^2^) is the surface area of the specimen; *V* (m^3^) is the leachate volume; *t* (day) is the leaching time duration; and the mass fraction of element (*i*) in the initial waste glass is represented by the term *f*_*i*_ (without unit).

As a result, it is possible to determine the leaching rates of elements as a function of time; hence, the leaching rates according to the experimental design method used in this study will be analyzed by producing three-dimensional surface plots with the influence of temperature and pH in different ranges.

## 3 Results and discussion

### 3.1 Experimental normalized leaching rates of RSM via BBD design

The BBD matrix produced with randomly selected 17 sets of experimental runs, along with the response and predicted results for the normalized leaching rate of Na (Na-NLR) and the normalized leaching rate of Si (Si-NLR) from the interaction of the three leaching factors, is shown in Table 5. After fitting the experimental data to a reduced quadratic model, the statistical significance and validity of the model were examined using the analysis of variance (ANOVA) method described in Section 3.2. The model Eqs 4, 5 are stated with the experimental values of the input parameters (A, B, and C), their interaction (AB, AC, BC), and quadratic (A^2^, B^2^, C^2^) components as a function of the responses Y_1_ and Y_2_, where Y_1_ is the response of the Na-NLR and Y_2_ is the response of the Si-NLR. The Eqs 4, 5 were displayed according to the modified model for the parameters with *p*-value less than 0.0500 and they expressed in terms of coded factors can be utilized to predict the response at different levels of each factor. By default, the high levels of the factors are coded as +1, and the low levels are coded as −1. The coded equation is useful for identifying the relative impact of the factors by comparing the factor coefficients. The synergistic impact of the term on the response is shown by the positive sign, whereas the antagonistic impact is indicated by the negative sign. To find the best fit, the sequential F-test, lack-of-fit test, and other adequacy metrics were used to analyze the statistical significance of the terms in each regression equation.

**TABLE 5:** Results of BBD with actual and RSM predicted data.

Run	Independent variables			Dependent variables (Responses)			
	Factor A: Temp ℃	Factor B: pH	Factor C: Time, d	Na-NLR [g/(m^2^.d)]		Si-NLR [g/(m^2^.d)]	
				Actual	Predicted	Actual	Predicted
1	70	8	21	1.03E-02	1.02E-02	9.23E-03	9.12E-03
2	50	8	14	8.46E-03	7.94E-03	5.14E-03	5.13E-03
3	70	10	14	1.72E-02	1.74E-02	1.30E-02	1.32E-02
4	50	10	21	7.59E-03	8.13E-03	7.60E-03	7.76E-03
5	70	12	7	2.91E-01	2.95E-01	2.47E-01	2.52E-01
6	70	12	21	1.13E-01	1.09E-01	9.07E-02	8.51E-02
7	50	12	14	1.08E-01	1.05E-01	6.36E-02	6.61E-02
8	90	10	21	2.27E-02	2.24E-02	2.01E-02	2.14E-02
9	70	10	14	1.68E-02	1.74E-02	1.35E-02	1.32E-02
10	90	12	14	2.30E-01	2.42E-01	1.91E-01	1.91E-01
11	70	10	14	1.78E-02	1.74E-02	1.27E-02	1.32E-02
12	90	10	7	5.35E-02	5.01E-02	5.72E-02	5.62E-02
13	70	10	14	1.66E-02	1.74E-02	1.37E-02	1.32E-02
14	50	10	7	2.12E-02	2.14E-02	1.68E-02	1.58E-02
15	90	8	14	2.19E-02	2.24E-02	1.74E-02	1.70E-02
16	70	8	7	2.15E-02	2.24E-02	1.56E-02	1.66E-02
17	70	10	14	1.78E-02	1.74E-02	1.30E-02	1.32E-02

The data in Table 5 shows how closely the experimental and predicted normalized leaching rates of Na and Si were related. The relative consistency of the data further confirms the model’s applicability (Teimouri, 2020). The statistical parameters (ANOVA) related to confirming the correspondence between the experimental and predicted normalized leaching rates will be further discussed in Section 3.2.

$$\left(Y_{1}\right)={\mathrm{Log}}_{10}\left(\text{Na}-\text{NLR}\right)=-\;1.76+0.2024A+0.5373B\;-\;0.1937C+0.4118B^{2}+0.0688C^{2}$$ (4)

$$\left(Y_{2}\right)={\mathrm{Log}}_{10}\left(\text{Si}-\text{NLR}\right)=+4.20230+0.005444A\;-1.49274B\;-\;0.058357C\;-\;0.000198\text{AC }-0.003694\text{BC}+0.000069A^{2}+0.090739B^{2}+0.002967C^{2}$$ (5)

### 3.2 Variance analysis (ANOVA)

**FIGURE 2:**
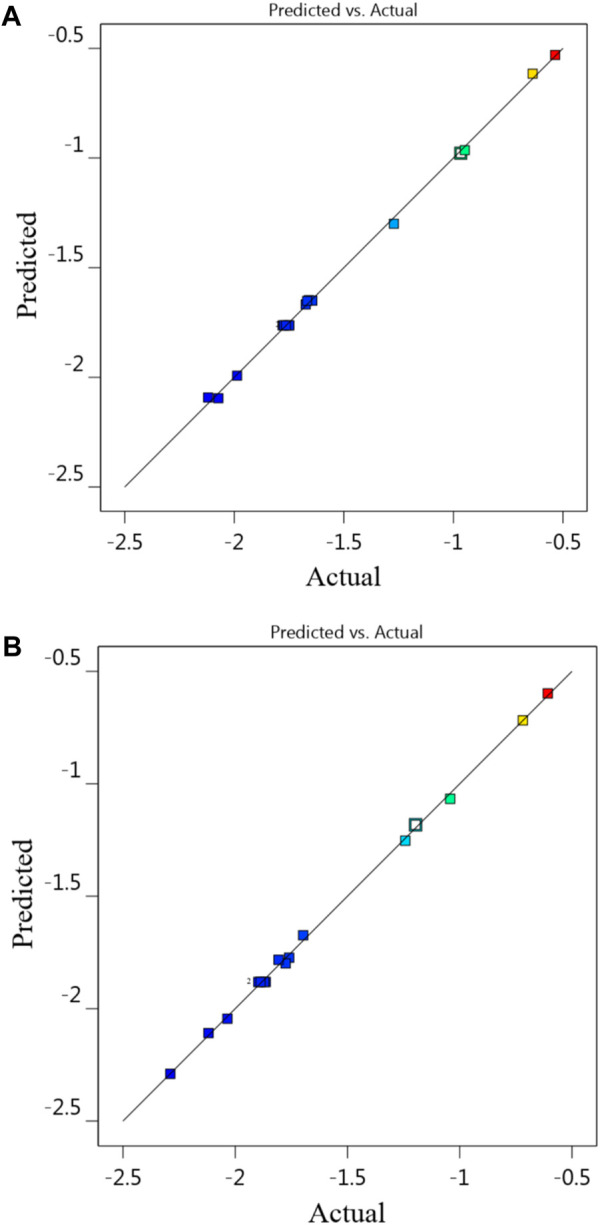
The diagnostic plot of predicted vs. actual values for **(A)** Na-NLR and **(B)** Si-NLR.

The model accuracy in this work is investigated by the coefficients of determination-R^2^ and adjusted-R^2^, the results of the analysis for Na-NLR and Si-NLR responses are indicated in Table 6. As seen from Table 6, for the response of Na-NLR, the predicted *R* ^2^ of 0.9912 is in reasonable agreement with the adjusted *R* ^2^ of 0.9957, i.e., the difference is less than 0.02, and for the response of Si-NLR, the predicted *R* ^2^ of 0.9872 is in reasonable agreement with the adjusted *R* ^2^ of 0.9976, i.e., in this response as well as Na-NLR, the difference is also less than 0.02. An adequate precision of 82.669 and 94.612 for responses indicates an adequate signal. According to the findings, the model was appropriate for the experimental data (Mahmoudiani et al., 2022). Figure 2 displays the validation of the predicted response values with the actual response values for Na-NLR and Si-NLR.

**TABLE 6:** Variance analysis (ANOVA) results for the response quadratic models.

Parameter	Na-NLR	Si-NLR
Standard deviation	0.0316	0.0236
Mean	−1.53	−1.63
Coefficient of variance (CV, %)	2.06	1.45
Coefficient of determination (R^2^)	0.9970	0.9988
Adjusted R^2^	0.9957	0.9976
Predicted R^2^	0.9912	0.9872
Adequate precision	82.0868	97.5829

**FIGURE 3:**
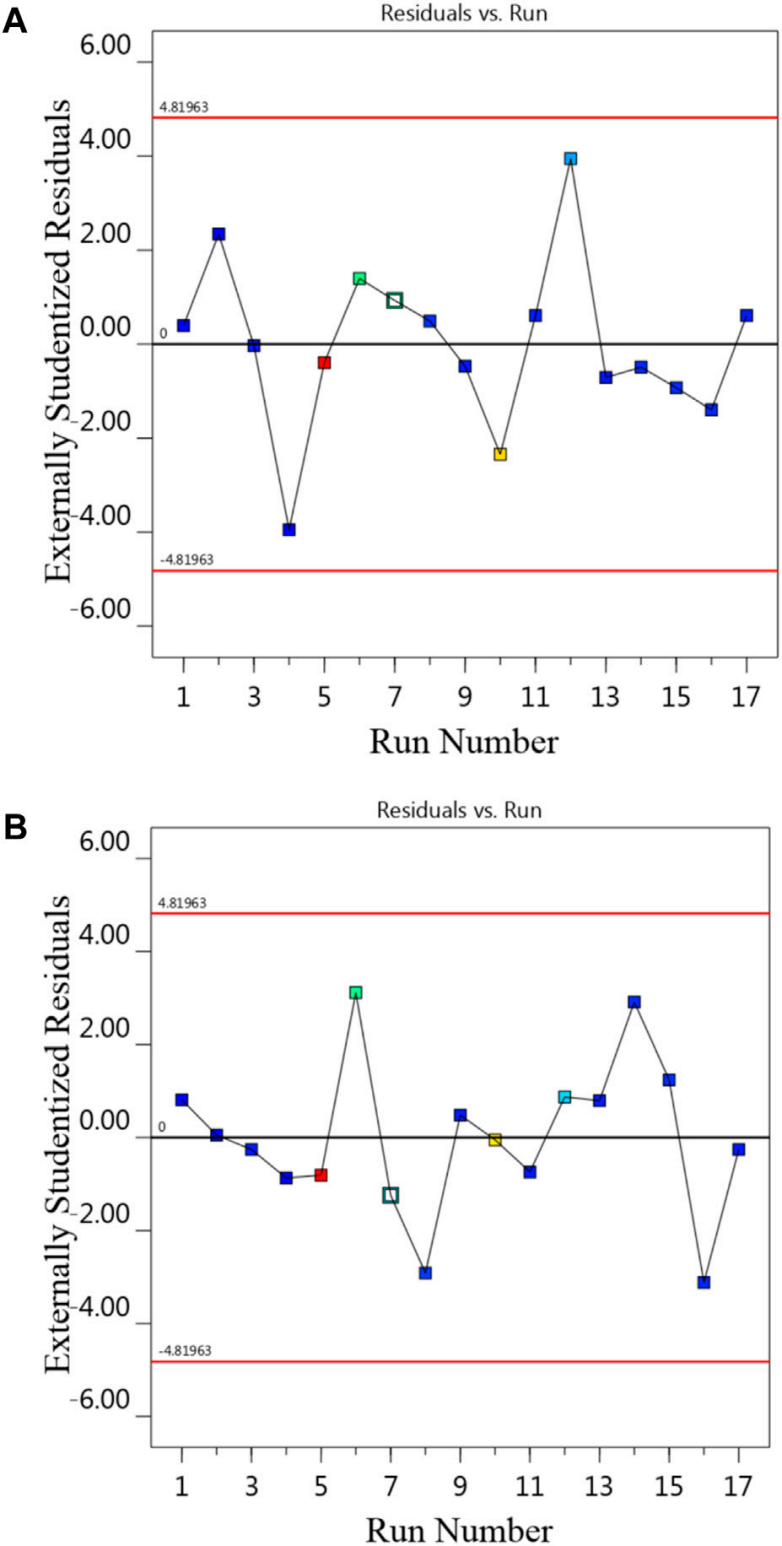
The diagnostic plot of residuals vs. run for **(A)** Na-NLR and **(B)** Si-NLR.

Tables 7, 8 illustrate the ANOVA results for Na-NLR and Si-NLR, respectively. According to Tables 7, 8, the F-values of 674.12 and 773.27 for Na-NLR and Si-NLR, respectively, imply the models are significant. In both models, there is only a 0.01% chance that an F-value this large could occur due to noise. Also, in Tables 7, 8, significant and insignificant model terms can be seen. It is perfectly clear that *p*-values less than 0.0500 and greater than 0.0500 indicate that model terms are significant and insignificant, respectively. For the response of Na-NLR, the F-value and *p*-value for “Lack of Fit” are 5.95 and 0.0589, and for the response of Si-NLR, they are 5.66 and 0.0636, respectively, which reveal that the “Lack of Fit” is not significant. It is not far from expected that to have a fitting model, there should be an insignificant lack of fit (Abazarpoor et al., 2013; Astutiningsih et al., 2022; Hamza et al., 2022). The diagnostic plot of residuals vs. run order of experiments for responses is represented in Figure 3, demonstrating that the residuals are random in nature and don’t display any pattern with run order and revealing that there is no noticeable pattern or unusual structure associated with the data (Sahoo and Mishra, 2014). Externally studentized residuals based on a deletion method are the default due to being more sensitive for finding problems with the analysis. It is obvious from Figure 3 that all points are inside the red lines (+4.81963 and −4.81963 externally studentized residuals), which ensures that the model fits effectively.

**TABLE 7:** Variance analysis (ANOVA) for the response of Na-NLR quadratic models.

Source	Sum of squares	df	Mean square	F-value	*p*-value	
Model	3.70	9	0.4107	674.12	< 0.0001	significant
A	0.3278	1	0.3278	538.00	< 0.0001	significant
B	2.31	1	2.31	3,791.48	< 0.0001	significant
C	0.3002	1	0.3002	492.82	< 0.0001	significant
AB	0.0018	1	0.0018	2.95	0.1295	not significant
AC	0.0014	1	0.0014	2.22	0.1795	not significant
BC	0.0022	1	0.0022	3.55	0.1016	not significant
A^2^	0.0014	1	0.0014	2.27	0.1754	not significant
B^2^	0.7107	1	0.7107	1,166.64	< 0.0001	significant
C^2^	0.0194	1	0.0194	31.82	0.0008	significant
Residual	0.0043	7	0.0006			
Lack of fit	0.0035	3	0.0012	5.95	0.0589	not significant
Pure error	0.0008	4	0.0002			
Cor total	3.70	16				

**TABLE 8:** Variance analysis (ANOVA) for the Response of Si-NLR quadratic models.

Source	Sum of squares	df	Mean square	F-value	*p*-value	
Model	3.78	9	0.4203	773.27	< 0.0001	significant
A	0.4811	1	0.4811	885.15	< 0.0001	significant
B	2.34	1	2.34	4,301.98	< 0.0001	significant
C	0.2669	1	0.2669	490.98	< 0.0001	significant
AB	0.0007	1	0.0007	1.23	0.3043	not significant
AC	0.0031	1	0.0031	5.66	0.0489	significant
BC	0.0107	1	0.0107	19.68	0.0030	significant
A^2^	0.0032	1	0.0032	5.82	0.0466	significant
B^2^	0.5547	1	0.5547	1,020.48	< 0.0001	significant
C^2^	0.0890	1	0.0890	163.72	< 0.0001	significant
Residual	0.0038	7	0.0005			
Lack of fit	0.0031	3	0.0010	5.66	0.0636	not significant
Pure error	0.0007	4	0.0002			
Cor total	3.79	16				

**FIGURE 4:**
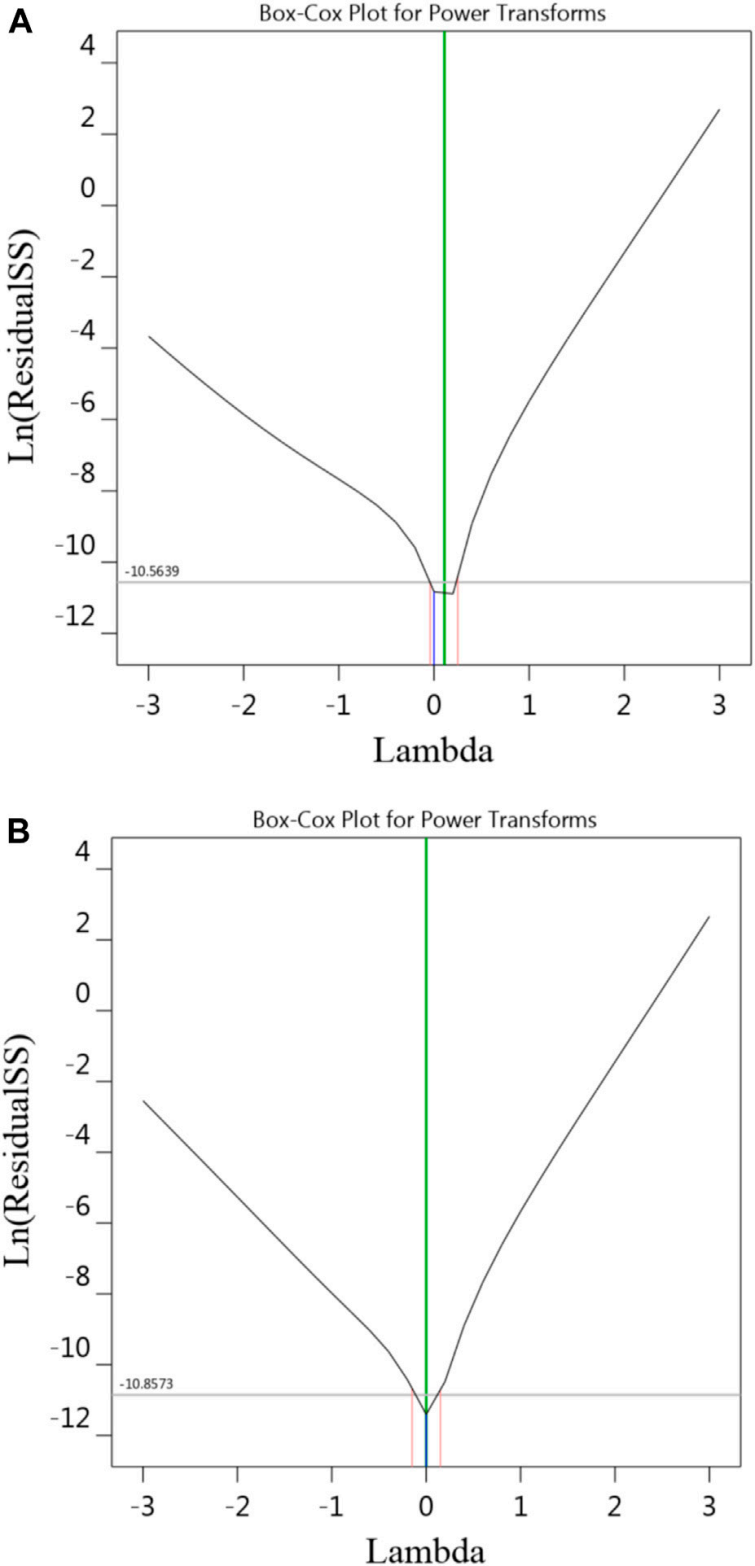
The diagnostic plot of Box-Cox for transformation, **(A,B)** are related to Na-NLR and Si-NLR respectively.

Given that it is an important assumption for statistical data to follow a normal distribution, the Box–Cox transformation was used to analyze the data obtained in this research. Figure 4 indicates the plot of the Box-Cox for Na-NLR and Si-NLR. As can be seen from Figure 4, the values of Lambda, which symbolizes the power applied to the response values obtained for Na-NLR and Si-NLR, are within the 95% confidence interval (Amdoun et al., 2010; Oroumei and Naebe, 2017; Freya and Senthil, 2022). It can be concluded that there is no need to change the response transformation because the models are in the optimum region.

### 3.3 Effects of the factors on the responses of the Na-NLR and the Si-NLR

**FIGURE 5:**
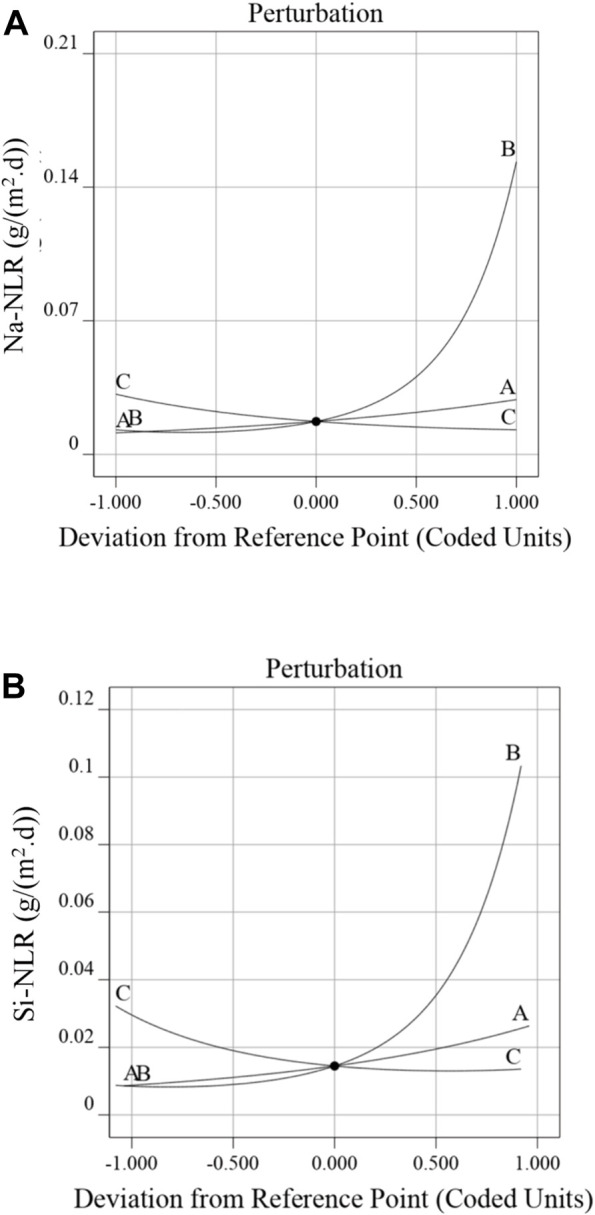
Perturbation plot displaying the effect of factors A, B, and C on the **(A)** Na-NLR and **(B)** Si-NLR.

The perturbation plots for the Na-NLR (a) and Si-NLR (b) models are shown in Figure 5. These plots provide an outline view of the normalized leaching rates of Na and Si and indicate how these responses alter when any independent variable deviates from the reference value while keeping all other variables constant (Oroumei and Naebe, 2017). It is perfectly evident from Figure 5 that the pH of leachate (factor B) has a negative impact on the durability of PWRHLW-BSG-1simulated waste glass to leaching, and it should also be noted that according to the perturbation plots, the effect of pH on sodium leaching rate was greater than silicon leaching rate. Factors A (temperature) and C (leaching time) have little positive and negative effects, respectively, on the Na-NLR and Si-NLR. It cannot be ruled out that the pH of leachant produces a higher effect on the investigated responses as compared to temperature and leaching time factors.

**FIGURE 6:**
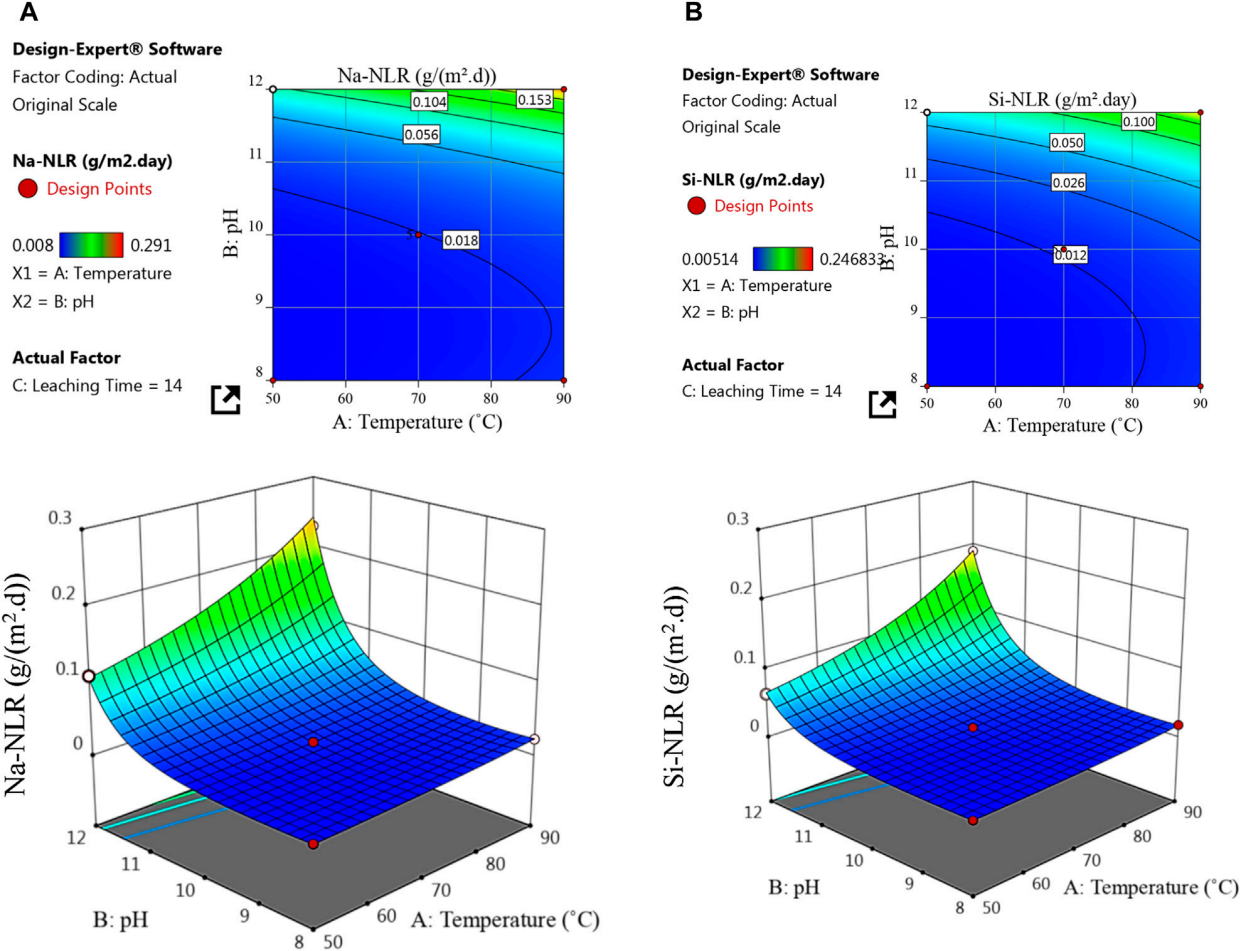
2D and 3D response surface plots for the effect of temperature and pH on the **(A)** Na-NLR and **(B)** Si-NLR during period of 14 days.

**FIGURE 7:**
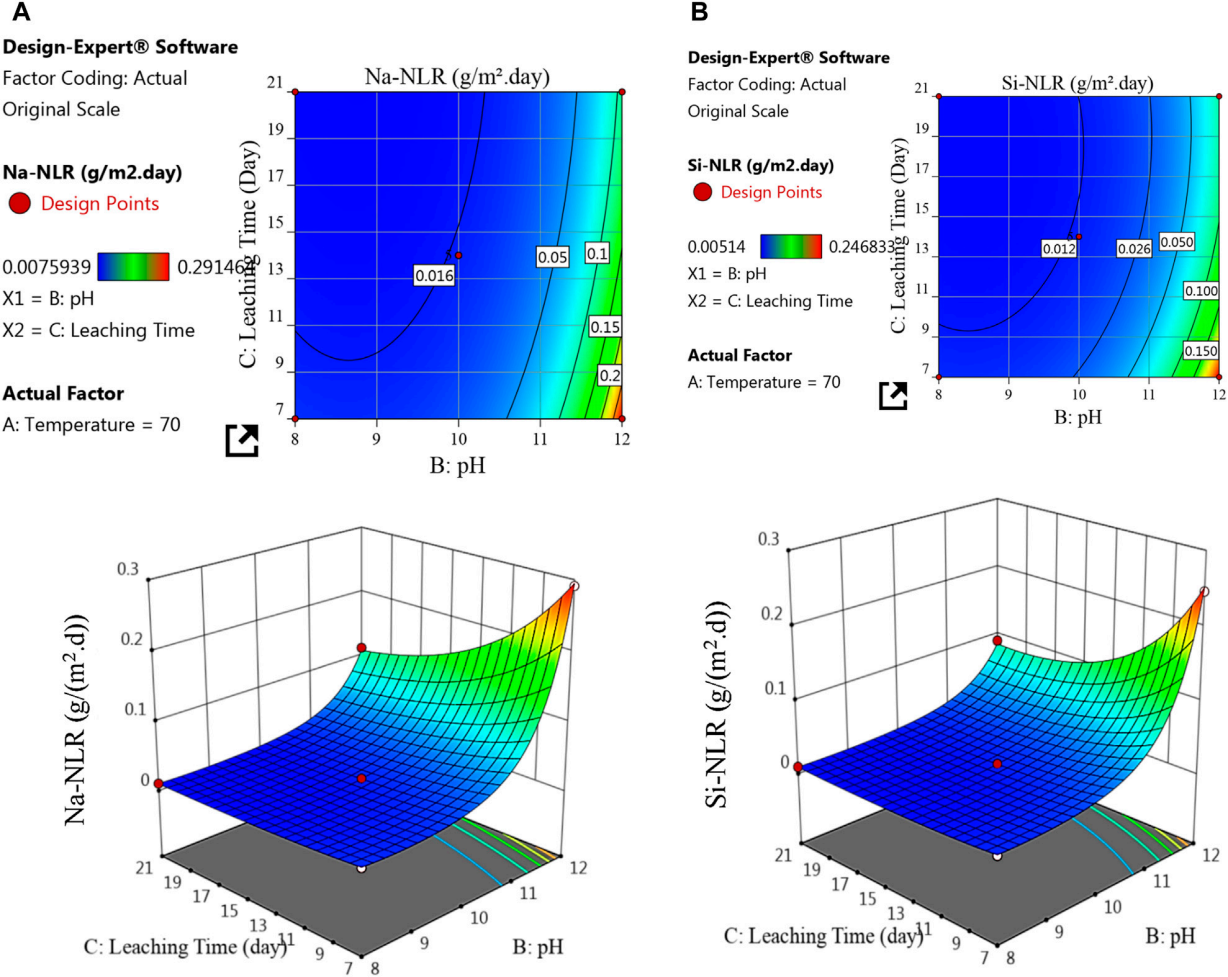
2D and 3D response surface plots for the effect of pH and leaching time on the **(A)** Na-NLR and **(B)** Si-NLR at temperature of 70°C.

Two and three-dimensional surface plots were created using quadratic polynomial model equations (Eqs 4, 5) to visualize the correlation between the Na-NLR and Si-NLR, which are the dependent variables, and the leaching conditions, which are the independent variables (Figures 6, 7). As can be seen from Figure 6, the period of leaching time was set at the center point (14 days), and the Na-NLR (a) and Si-NLR (b) vary with pH and temperature changes. According to the 2D and 3D surface plots in Figure 6, the Na-NLR and Si-NLR increase with increasing pH from 8 to 12. Especially at pH 12, this increase in leaching rates is more severe for both elements. Also, it is worth mentioning that the Na element in PWRHLW-BSG-1 simulated waste glass over a period of 14 days is more sensitive to pH changes and has been leached more than the Si element. As the temperature of the leachate throughout this time period rises from 50°C to 90°C, the normalized leaching rates of Na and Si from the PWRHLW-BSG-1 increase, but they do so slowly. As a result, with the simultaneous increase in pH and temperature, the leaching rates of both elements increase, so, during a period of 14 days, the maximum leaching rates for Na and Si have been calculated at pH 12 and temperature 90°C, that is, Na-NLR is equal to 0.23 g/(m^2^. d) and Si-NLR is equal to 0.19 g/(m^2^. d). It is also better to mention that the slope of the normalized leaching rate as a function of temperature is therefore less steep than the slope of the normalized leaching rate as a function of pH, i.e., the Na-NLR and Si-NLR are more sensitive to the pH effect compared to the temperature effect.

Due to the sorption of H^+^, OH^−,^ and H_2_O on the glass surface, pH plays an important role in the glass dissolving reaction. For usage in acidic, neutral, and alkaline solutions, various parameter values for the pH dependence have been recommended (Köhler et al., 2003; Zapol et al., 2013; Neeway et al., 2018). It is important to keep in mind that a lot of minerals and glasses show a V-shaped dependency in the dissolving rate as a function of pH, meaning that the rate is often high in acidic solutions, approaches a minimum in the range of pH values that are close to neutral, and rises as the alkalinity of the solution’s contents rises (Brantley, 2008; Kim et al., 2011; Inagaki et al., 2012). The slope of glass dissolving as a function of pH is not the same for acidic and alkaline solutions, but this known behavior may be approximated by simply altering the pH dependence coefficient (Jeong and Ebert, 2002; Pierce et al., 2008; Vienna et al., 2018), when circumstances shift from alkaline to acidic (Inagaki et al., 2013; Kweinor Tetteh et al., 2021).

The dissociation of H_4_SiO_4_ into H_3_SiO_4_^1–^ and H_2_SiO_4_^2–^ species has been demonstrated to significantly improve the solubility of silica over pH 9. As a result, it may be anticipated that Si release rates and, therefore, alkali ion releases may rise as a result of ion exchange activities (Cassingham et al., 2015). The process of ion exchange is a significant reaction since it enables the disintegration of the glass network into the aqueous phase. This happens as a result of the local pH being raised by the loss of H^+^ from the aqueous phase, which in turn produces OH^−^ ions that can attack the structure of glass directly (McGrail et al., 2001; Pierce et al., 2008). It is generally accepted that this mechanism involves the inter-diffusion of H (as H^+^ or H_3_O^+^) in solution and network-modifying cations in the glass, while the network-forming components’ ionic covalent bonds (Si-O-Si, Si-O-Na, etc.) suffer hydrolysis and are attacked by OH^−^ through nucleophilic nucleation. The breaking of the Si-O bonds and the separation of Si are thought to be the rate-limiting steps in this reaction (Cassingham et al., 2015). Therefore, according to these presented arguments, it is concluded that the leaching mechanism of PWRHLW-BSG-1 simulated waste glass has been affected by the release of H_3_SiO_4_^1–^ and H_2_SiO_4_^2–^ during the leaching process. Table 4 confirms this conclusion because all the pHs after leaching are lower than the pHs before leaching. H_3_SiO_4_^1–^ and H_2_SiO_4_^2–^ ions increase the H^+^ species in the solution and further decrease the pH.

Also, to justify the effect of temperature, as mentioned above, raising the temperature increased each element’s normalized dissolution rate. According to the empirical Arrhenius equation (Jeong and Ebert, 2002; Pierce et al., 2008; Neeway et al., 2018; Vienna et al., 2018), a description of the observed temperature dependence on the dissolving rate is provided. It is obvious that, variations in temperature and pH influence the process of glass modification because they vary the activation energies of elements at various pHs (Inagaki et al., 2012; Cassingham et al., 2015).

The leaching behavior of Na and Si with respect to temperature and pH changes in the PWRHLW-BSG-1 simulated waste glass investigated in this study, along with the leaching behavior of Na and Si elements and other important elements involved in the structure of glasses in the matrices of different waste glass, has been compared, so that these matrices with different leaching models in temperature and pH under similar conditions have been tested. The results show that the pattern of changes in the leaching rates of the elements investigated in this research is very similar to the pattern of changes in the leaching rates of elements in glasses of AFCI, ISG, SON68 (Neeway et al., 2018), LAWA44, LAWB45, LAWC22, SRL202 (Pierce et al., 2008), MT25, MT30 (Cassingham et al., 2015), a complex borosilicate glass (Abraitis et al., 1997), LD6-5412 (McGrail et al., 1997), P0798 (Inagaki et al., 2012), and ISG (Backhouse et al., 2018).

Figure 7 depicts the interaction effects of pH and leaching time on the (a) Na-NLR and (b) Si-NLR at a fixed center point of temperature (70°C). As can be observed from the 2D and 3D surface plots in Figure 7, at the temperature of 70°C with an increasing pH from 8 to 12, the Na-NLR and Si-NLR increase, but factor C of the leaching time has a little negative effect on the Na-NLR and Si-NLR, so that, after the glass was affected by the leaching process and the Na and Si began to be released, the leaching rates of elements gradually reached an approximately constant value over time. As a result, with the simultaneous increase in pH and leaching time, the leaching rates of both elements increase in general, but this increase has a lower intensity compared to the effect of the simultaneous increase in temperature and pH on the leaching rates.

The comparison of the leaching behavior of Na and Si as a function of time in the PWRHLW-BSG-1 with the leaching behavior of Na and Si elements and other important elements in the various waste glasses, so that these glasses have been tested with different leaching models in pH and leaching time under similar conditions, gave the result that the pattern of changes in the leaching rates of the elements studied in this work is very similar to the pattern of changes in the leaching rates of elements in other different waste glasses. For example, glasses of LD6-5412 (McGrail et al., 1997), a simple five-component borosilicate glass (Knauss et al., 1989), P0798 (Inagaki et al., 2012), LAWA44 (Pierce et al., 2008), DG2B (Kim et al., 2011), P0798 (Inagaki et al., 2006), CSG (Jeong and Ebert, 2002), and a natural glass (Wolff-Boenisch et al., 2004) were compared.

From a kinetic perspective, transport or chemical interactions at the interface control how quickly glasses dissolve. Surface reaction control is indicated by a constant leachate concentration throughout time in the leaching tests. The leached glass is vulnerable to leachant attack, which causes partial glass disintegration and changes to the surface. Some glass constituents leak into the leachate, and other glass and leachate constituents bind to the surface of the glass to produce a precipitated layer. It comprises a crystal and/or amorphous collection with impacts on the progression of the leaching process and corrosion growth. Based on these principles of glass leaching kinetics and considering that the Na-NLR and Si-NLR in the PWRHLW-BSG-1 remain approximately constant over time, and since the PCT test was done in a closed system, it can be considered that the leaching of the PWRHLW-BSG-1 follows four steps: the diffusion of water substances into the glass structure (stage I), ion exchange with protons (stage II), hydrolysis of network-modifying species in the PWRHLW-BSG-1 structure (stage III), and the formation of a precipitated layer on the surface of the glass due to the saturation of the solution with leached species in a closed system (stage IV). For interest, in the work (Luo et al., 1997), the compositional changes in the surface layer, surface layer precipitation, surface layer pitting corrosion, and surface layer break and spallation were detailly examined.

A similar conclusion has been drawn related to stage IV of PWRHLW-BSG-1 leaching in a static test (Frankel et al., 2018). In that test, as time passes, the concentration of components in the leachate rises, and the leach rate progressively falls as the chemical affinity for dissolution decreases. The soluble capacity of secondary phase precipitates, particularly for incongruent dissolution, may have an impact on the pace of dissolution as dissolved components accumulate. The concentration of leachate in solution approaches saturation as a result of a buildup of the dissolved molecules in bulk solution and a consequent reduction in under saturation.

Another of our possible hypotheses regarding the reason for the constant Na-NLR and Si-NLR in the PWRHLW-BSG-1 over time is in accordance with the findings of research work (Inagaki et al., 2012). It is stated that the relatively higher rate of dissolution at the beginning of the test period may have been brought on by the initial glass specimen’s larger surface area, which was originally rough due to polishing. As the glass dissolves, the roughness becomes smoother, resulting in a constant dissolution rate for the duration of the test period, which extends above 20 h. Anyway, in order to check the correctness of the proposed hypothesis, it needs future analyses related to PWRHLW-BSG-1 simulated waste glass.

### 3.4 Experimental validation of the BBD model

To evaluate the employed model to study the leaching behavior of PWRHLW-BSG-1 simulated waste glass in this research, as well as to ensure the accuracy of the results obtained from leaching Na and Si, in addition to the suggested points of the model, six confirmation experiments were conducted in parallel with different parameters other than the points designed by the model. For this purpose, the laboratory results obtained for these 6 tests in the “Post Analysis—Confirmation” section of Design-Expert software, according to Tables 9, 10, for Na-NLR and Si-NLR, respectively, have been compared with statistical data within the predicted range at a 95% confidence level. As can be seen from Tables 9, 10, the actual leaching rates obtained for Na and Si are reasonably close to the predicted values and are located within 95% PI (Prediction Interval) low and 95% PI (Prediction Interval) high intervals (Oroumei and Naebe, 2017; Enyoh et al., 2022; Freya and Senthil, 2022). Hence, it can be concluded that consistency between the data verifies the model’s potential for prediction that was employed in this work and reveals that the experimental result is substantially close to the desired value.

**TABLE 9:** Confirmatory values of BBD analysis results for Na-NLR.

Response	Na-NLR [g/(m^2^.d)]					
Number of experiment according to Table 3	1	2	3	4	5	6
Predicted mean	1.29E-02	3.16E-02	1.13E-02	2.87E-02	1.29E-02	1.53E-01
Predicted median	1.29E-02	3.15E-02	1.13E-02	2.87E-02	1.29E-02	1.53E-01
Std Dev	7.34E-04	1.79E-03	6.43E-04	1.63E-03	7.35E-04	8.72E-03
95% PI low	1.10E-02	2.69E-02	9.65E-03	2.45E-02	1.10E-02	1.31E-01
Actual data	1.12E-02	2.96E-02	1.08E-02	2.50E-02	1.13E-02	1.69E-01
95% PI high	1.51E-02	3.69E-02	1.32E-02	3.35E-02	1.51E-02	1.79E-01

**TABLE 10:** Confirmatory value of BBD analysis results for Si-NLR.

Response	Si-NLR [g/(m^2^.d)]					
Number of experiment according to Table 3	1	2	3	4	5	6
Predicted mean	8.74E-03	2.80E-02	7.97E-03	2.47E-02	1.21E-02	1.05E-01
Predicted median	8.73E-03	2.80E-02	7.96E-03	2.46E-02	1.21E-02	1.05E-01
Std Dev	4.70E-04	1.51E-03	4.28E-04	1.32E-03	6.49E-04	5.66E-03
95% PI low	7.53E-03	2.41E-02	6.86E-03	2.12E-02	1.04E-02	9.08E-02
Actual data	8.31E-03	2.49E-02	6.90E-03	2.28E-02	1.08E-02	1.22E-01
95% PI high	1.01E-02	3.24E-02	9.23E-03	2.86E-02	1.40E-02	1.22E-01

However, the authors advise that in future research, the PWRHLW-BSG-1 be subjected to experimental and statistical analysis with leaching tests other than PCT and over a wide range of time and temperature intervals, as well as in environments with acidic and alkaline pH with a pH difference of 1. It is also better to be notified that the leaching behavior of two other important elements, boron and strontium, in PWRHLW-BSG-1 is currently being analyzed in another research work. Nevertheless, the results obtained under the new conditions and the comparison with the previous results, as well as the evaluation of the potential of the model under variable circumstances and the comparison of the leaching behavior of B and Sr elements with Na and Si, allow for more decisive decisions to be made regarding the PWRHLW-BSG-1 as a matrix for the immobilization of spent nuclear fuel components.

## 4 Conclusion

The investigation of the leaching behavior of Na and Si elements as a function of time, pH, and temperature in HLW borosilicate glass simulated from waste of a 1000 MWe class PWR reactor using RSM and BBD leads to the following conclusion:

1. Experiments using the PCT leaching test were conducted at three different temperatures of 50, 70, and 90°C (Factor A), under alkaline conditions at pH (Factor B) 8, 10, and 12, and during periods of 7, 14, and 21 days (Factor C). The experimental and predicted normalized leaching rates of Na and Si obtained through 17 sets of experimental runs produced by BBD were related. The relative consistency of the data further confirmed the model’s applicability.
2. The results of statistical analysis (ANOVA) for Na-NLR and Si-NLR indicated that the effects of the individual variables and some of the interactions between the variables were statistically significant. The diagnostic plots of predicted vs. actual values and residuals vs. run for Na-NLR and Si-NLR confirmed the validation of the data and revealed that there is no noticeable pattern or unusual structure associated with the data. The values of Lambda obtained through the Box–Cox transformation are within the 95% confidence interval.
3. According to the 2D and 3D surface plots, while the period of leaching time was set at the center point (14 days), with the simultaneous increase in pH and temperature, the leaching rates of both elements Na and Si increased; nevertheless, the slope of the normalized leaching rate as a function of temperature is therefore less steep than the slope of the normalized leaching rate as a function of pH. It is obvious that variations in temperature and pH influence the process of glass modification because they vary the activation energies of elements at various pHs.
4. When the temperature of 70°C was fixed at a center point, the 2D and 3D surface plots indicated that with an increasing pH, the Na-NLR and Si-NLR increased, but factor C of the leaching time had a little negative effect on the rates. So that, after the glass was affected by the leaching process and the Na and Si began to be released, the leaching rates of the elements gradually reached an approximately constant value over time. Hence, with the simultaneous increase in pH and leaching time, the leaching rates of both elements increase in general, but this increase has a lower intensity compared to the effect of the simultaneous increase in temperature and pH on the leaching rates.
5. The comparison of the leaching behavior of Na and Si in the PWRHLW-BSG-1 with the leaching behavior of the mentioned elements and other important elements in the various waste glasses, so that these glasses have been tested with different leaching models in temperature, pH, and leaching time under similar conditions, gave the result that the pattern of changes in the leaching rates of the elements studied in this work is very similar to the pattern of changes in the leaching rates of elements in other different waste glasses.
6. The experimental validation of the BBD model showed that the actual leaching rates obtained for Na and Si are reasonably close to the predicted values and are located within 95% PI low and 95% PI high intervals.

## Data Availability

The original contributions presented in the study are included in the article/Supplementary material, further inquiries can be directed to the corresponding authors.

## Footnotes

1 The F-value in one way ANOVA is a tool to help you answer the question “Is the variance between the means of two populations significantly different?” The F-value in the ANOVA test also determines the *p*-value; The *p*-value is the probability of getting a result at least as extreme as the one that was actually observed, given that the null hypothesis is true.
